# State of art and science advances on nutrition and nutrigenetics in nutrition-related non-communicable diseases in Middle East

**DOI:** 10.1186/s12967-015-0390-7

**Published:** 2015-02-01

**Authors:** Laura Soldati, Abdelhamid Kerkadi, Paul Amuna, Annalisa Terranegra

**Affiliations:** Department of Health Sciences, Università degli Studi di Milano, Milan, Italy; Department of Health Sciences, Qatar University, Doha, Qatar; Research Section of the Primary Health Care Corporation, Doha, Qatar; Translational Medicine Department, Sidra Medical and Research Center, Doha, Qatar

Non-Communicable Diseases (NCDs) account for more than 60 per cent of global deaths annually [1] and this is estimated to increase exponentially by 2025 if present trends continue. In the last decades, Science of Nutrition became relevant, not only for the body fitness and aesthetics, but also for the healthy. In fact, Nutrition-Related Non-Communicable Diseases (NR-NCDs) are pathologies like diabetes, hypertension, obesity, cardiovascular disease, metabolic syndrome and cancers, where nutrition has a major role in both prevention and therapy.

Moreover, NR-NCDs are caused by the complex relations between genetic and environmental background. Nutrigenetics and epigenetics are sciences that study the interactions between dietary components and the genome as well as resulting changes in gene expression, proteins and metabolite activities. A growing number of studies are focusing on food effects on gene expression modification. Microbiome composition, methylome and miRNA analysis are the arguments pointed by nutrigenetics and epigenetics. Recently, advanced technologies (as next generation sequencing, high-density micro-arrays, mass spectrometry, bioinformatics) allowed us to easily approach these sciences [2].

In few decades, the Middle East has been witnessing significant changes in food habits, losing traditional healthy diet, rich in vegetable proteins, fibers, minerals and vitamins, in the favor of a more industrial diet, consisting of preprocessed foods, sugars, fats, animal products, saturated- and trans- fatty acids [3]. In this landscape, NR-NCDs increased exponentially and are estimated to account for 69% of total deaths in Qatar [4]. Moreover, the Qatari population has a unique genetic background because of the migration history and the high rate of consanguineous marriages (54%), resulting in high prevalence of NR-NCDs [5].

The nutritional habits and life-style were demonstrated to improve the resolution and frequency of NR-NCDs, but poor information is available about diet in terms of macro and micro components and caloric intake in the Middle East countries. New tools are needed to collect more precise estimates of these population dietary habits and to characterize their diets in terms of macro- and micro-nutrients. Moreover, ad hoc designed applications for smartphones could be developed to promote the subject compliance to personalized diet and the dialogue between patients and healthcare professionals.

Nutrigenetics and nutrigenomics sciences are just starting to address NR-NCDs in Qatar, so researcher interest and institution cooperation are called to join this mission.
